# Simulation of the Penetration Process of 7xxx Aluminum Alloy Laminates with Different Configurations

**DOI:** 10.3390/ma18102357

**Published:** 2025-05-19

**Authors:** Qunjiao Wang, Shuhan Zhang, Meilin Yin, Hui Zhang, Xinyu Liu, Ruibin Mei, Fuguan Cong, Yunlong Zhang, Yu Cao

**Affiliations:** 1 Key Laboratory of Electromagnetic Processing of Materials, Ministry of Education, Northeastern University, Shenyang 110819, China; shuhan202202@163.com (S.Z.); yinmeilin0303@163.com (M.Y.); hzhang@epm.neu.edu.cn (H.Z.); liuxinyu0016@163.com (X.L.); 2 State Key Laboratory of Rolling and Automation, Northeastern University, Shenyang 110819, China; meiruibin@neuq.edu.cn; 3 Northeast Light Alloy Co., Ltd., Harbin 150060, China; cfgdy2010@163.com (F.C.); zyl030202@126.com (Y.Z.); 4 International Joint Laboratory for Light Alloys, Ministry of Education, College of Materials Science and Engineering, Chongqing University, Chongqing 400044, China; caoyu928@cqu.neu.cn

**Keywords:** aluminum alloy, laminate, J-C constitutive models, numerical simulation, penetration

## Abstract

Aluminum alloy laminates have extensive applications in protective armor systems. A simulation-based approach was employed to investigate the anti-penetration performance of aluminum alloy laminates with different configurations. Experiments were carried out to study the mechanical properties of 7055 and 7075 aluminum alloys, and a J-C constitutive model was established for the 7055/7075 aluminum alloy laminate. Based on the J-C constitutive model, numerical simulation was performed to assess the anti-penetration performance of an aluminum alloy laminate with various configurations. Velocity curves during the projectile penetration process were obtained. The simulation results show that the four-layer laminate exhibits superior anti-penetration performance compared to the two-layer laminate. The four-layer laminate with the 7055/7075/7075/7055 configuration demonstrates optimal anti-penetration performance.

## 1. Introduction

In order to meet the continuous development needs of protective equipment, it is urgent to improve the anti-penetration performance of protective plates [1,2,3,4]. Compared with single-layer plates, laminates produced through the lamination process not only have finer grain sizes, but also contain multi-layer interfaces. This unique structural component enables these laminates to absorb more energy when the material fractures, thereby improving the anti-penetration performance of protective plates [5,6,7]. This laminated structure effectively combines the high strength of high-alloy Al alloys with the superior ductility of low-alloy Al alloys. Laminated metal composite structures represent a unique form of composites, consisting of alternating metallic layers or metal layers with discrete interfaces [8]. Such layered configurations play critical roles in enhancing fracture toughness [9,10], damage tolerance [11], impact behavior [12], damping capacity [13], and fatigue resistance, as well as improving the formability and ductility of otherwise brittle materials [14]. Previous studies have demonstrated that laminated aluminum plates can synergistically enhance the mechanical properties of their constituent materials [9,15,16,17,18]. For instance, Alic and Danesh revealed the emergence of R-curve behavior in aluminum laminates, a phenomenon absent in monolithic plates [17]. Research by B. Tekyeh showed significantly improved impact toughness in aluminum laminates compared to single-layer counterparts, with further enhancements achievable through increased layering and optimized interfacial adhesion [14]. In their fracture behavior studies of aluminum laminates, Jimenez et al. proposed that the toughening mechanism arises from energy dissipation through interfacial delamination, subsequent crack re-nucleation in adjacent layers, and plastic deformation of ductile layers. Their hypothesis suggests that interfacial delamination reduces crack-tip triaxiality and redistributes localized stresses, while crack re-nucleation contributes to toughening by dissipating energy for new crack initiation [13,16,17,19]. These findings collectively demonstrate that aluminum laminated structures are promising candidates for lightweight armor applications. Therefore, a comprehensive understanding of the high-velocity impact behavior of various aluminum laminate configurations is essential for advancing their practical implementation.

Scholars have conducted extensive research on aluminum alloy laminates. Semnan et al. [20] investigated the effect of the overlay rolling process (ARB) on the microstructure of laminates. Their research found that the ARB process generates a large number of dislocations in the material, forming substructures and subgrains. This ultimately leads to refined grain size and enhanced material yield strength. Song et al. [21] studied the effect of rolling passes on the properties of laminates, and their research found that as the number of rolling passes increases, the tensile strength of the AA1050/AA5052 laminates initially increases and then decreases. After the fourth rolling pass, the hard layer exhibits plastic instability, leading to a decrease in tensile strength. Liu et al. [22] studied the preparation of alumina (Al_2_O_3_) particle-reinforced laminates using the ARB process. Their research found that after 11 ARB cycles, the Vickers hardness of the laminate increased by 1.15 times and the tensile strength increased by 1.19 times.

Aluminum alloy is a suitable material for lightweight protective plates, due to its high strength, low density, and high ductility; 7xxx alloys exhibit particularly excellent mechanical properties, making them typical materials for protective plates with excellent anti-penetration performance [23,24,25]. In their study on the ballistic impact resistance of aluminum alloy materials, Christian et al. [26] employed experimental and numerical simulation methods to analyze the dynamic response of 7020-T6 aluminum alloy plates under penetration by different projectiles. They confirmed that conical projectiles induced sharp petal-shaped fractures with asymmetric fragment ejection upon perforation. Additionally, variations in projectile impact angles were found to significantly alter stress distribution in the projectile–target interaction zone, thereby modulating the fracture modes and failure pathways of the target plates. Teng’s team [27,28,29] demonstrated that laminated composite structures substantially enhanced resistance to flat-nosed projectile penetration compared to monolithic plates. This improvement was attributed to a transition in failure mechanisms from localized shear plugging to global tensile tearing, coupled with enhanced energy dissipation through interlayer delamination. Holmen et al. [30] systematically investigated the perforation resistance of thick aluminum plates under moderate-velocity impacts, concluding that material strength was not the dominant factor governing anti-penetration performance. Furthermore, Han et al. [31] combined experimental and computational approaches to evaluate the dynamic response of 2024-T351 aluminum alloy plates of varying thicknesses under blunt projectile impacts. Their work proved that incorporating the Lode angle parameter into fracture criteria significantly improved the predictive accuracy of ballistic resistance, providing a theoretical basis for engineering protective design.

In terms of J-C constitutive models, Shen et al. [32] established a J-C constitutive model for the 6061 alloy, revealing a negative correlation between the flow stress of the 6061 aluminum alloy and strain rate. Their research found that fracture strain decreases with increasing strain rate. Zhang et al. [33] established the J-C constitutive model for their 7075 aluminum alloy through experimental and simulation methods, and their research found that increasing the strain rate significantly increases strength. As the strain rate increases, necking at the specimen fracture becomes increasingly apparent. Wan et al. [34] investigated the dynamic compression of the 7A04-T6 alloy, and their research found that at strain rates of 1678 to 3621 s^−1^, the dynamic yield strength of the alloy increased by approximately 1.7% to 9.1%, and at strain rates of 1988 to 4001 s^−1^, the dynamic yield strength of the alloy increased by about 7.6% to 8.1%. Tan et al. [35] conducted room-temperature tensile tests on the 7050 alloy at strain rates ranging from 10^−3^ to 2900 s^−1^, and established both Johnson–Cook (J-C) and Khan–Liu constitutive models for the alloy. They found that the J-C model could effectively predict the stress–strain behavior at high strain rates. Chen et al. [36] also demonstrated that the J-C constitutive model exhibited good predictive accuracy for the room-temperature deformation behavior of the 7050 alloy.

At present, the exploration of the anti-penetration performance of 7xxx aluminum alloy laminates is relatively limited. In order to comprehensively evaluate the anti-penetration performance of 7xxx aluminum alloy laminates, this study employs the classical Johnson–Cook (J-C) constitutive model to characterize the dynamic mechanical behavior of the material [37], and further develops a J-C constitutive model specifically for AA7055/AA7075 aluminum alloy laminates [38]. Through simulation methods, the anti-penetration performance of 7055/7075 aluminum alloy laminates with different configurations was studied.

## 2. Materials and Methods

### 2.1. Materials

The experimental materials were two types of aluminum alloys, 7055 and 7075. Table 1 presents the chemical composition of the materials.

### 2.2. Experimental Methods

Hot rolling was selected for the preparation of bullets. The thickness ratio of layer 7055 to layer 7075 was maintained at 5:1. Before rolling, the surface of the bullet was treated with acetone to remove oil stains on the surface of the material, and then thoroughly cleaned with a steel brush. In order to prevent sliding between layers during the rolling process, four corners of the plate were punched and fixed with pure aluminum rivets, and the bullet was hot rolled after heating in a resistance furnace at 420 °C for 2 h to ensure complete preheating.

After roll bonding, the aluminum alloy laminates were subjected to heat treatment to improve the mechanical properties of the material. The heat treatment process included a solution treatment at a temperature of 470 °C for 1 h, followed by water quenching. The laminate was then aged. During the aging process, the temperature was maintained at 120 °C for 24 h.

**Figure 1:**
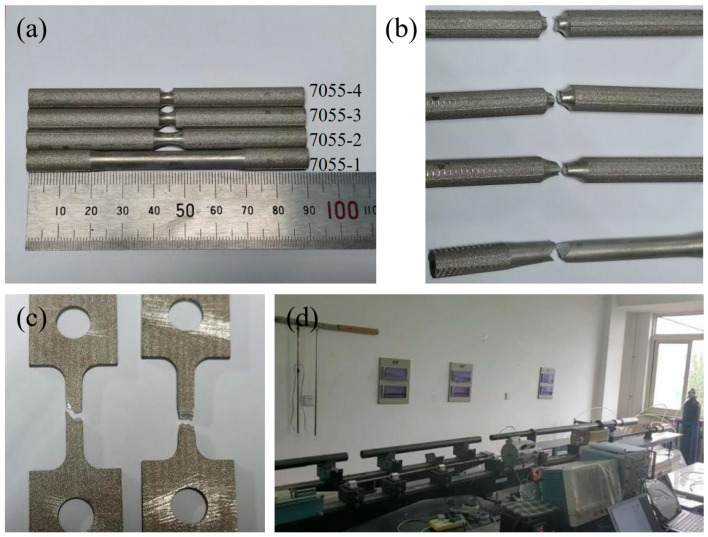
Tensile specimen of 7055 alloy and experimental setup. (**a**) Round-bar specimen before test; (**b**) fractured specimen; (**c**) fracture surface of 7055 alloy after high-temperature tension; (**d**) experimental setup of split Hopkinson pressure bar.

In order to establish a J-C constitutive model for the laminated plates, it was necessary to evaluate the tensile properties of each layer of alloy. The treatment methods for the 7055 alloy and the 7075 alloy were the same as those for laminates. Tensile experiment bars with different notch radii were prepared. The tensile experiments were carried out on a universal electronic testing machine at a displacement rate of 1.5 mm/min, and the strain was measured with an extensometer. The thickness of each tensile specimen was 3 mm, and the experimental temperatures were 100 °C and 200 °C. The tensile specimens of the 7055 alloy are shown in Figure 1a,b. The macroscopic morphology of the specimens after the tensile tests is shown in Figure 1c.

To assess the dynamic response behavior of the aluminum alloy at high strain rates, dynamic compression experiments at different strain rates were conducted using a split Hopkinson pressure bar (SHPB) experimental setup. Cylindrical specimens of Φ4 × 5 mm were prepared. SHPB is an experimental setup used to study the dynamic behavior of materials [39]. The basic principle involves generating and transmitting stress waves through a specimen to analyze its dynamic response. The setup typically includes a striker bar, an incident bar, a transmission bar, and the specimen. In an SHPB experiment, precise control of the impact, stress wave transmission, and measurement devices is crucial for obtaining accurate data on the dynamic behavior of the materials, particularly under high strain rates. The experimental setup is shown in Figure 1d.

## 3. Results

### 3.1. J-C Flow Stress Model

In order to meet the requirements of subsequent numerical simulations, this study established J-C constitutive models for the 7055 and 7075 aluminum alloys. The J-C constitutive model mainly consists of three parts: strain hardening, strain rate strengthening, and thermal softening of materials. This model comprehensively considers the relationships among flow stress, strain hardening, strain rate, and temperature.

The meanings of parameters used for the J-C constitutive model are shown in Table 2. The expression of the J-C model [37] is as follows:

(1) $$\sigma =\left(A+B\varepsilon^{n}\right)\left[1+C\ln\left(\dot{\varepsilon }/{\dot{\varepsilon }}_{0}\right)\right]\left[1-{\left(\left(T-T_{r}\right)/\left(T_{m}-T_{r}\right)\right)}^{m}\right]$$

Based on the results of the experiments for the 7055 and 7075 aluminum alloys, the material parameters for each layer in the laminate of the simulated model were determined as follows:

Tensile experiments were conducted at the reference strain rate of 10^−3^ s^−1^ and at room temperature, under which conditions the last two terms of Expression (1), $\left[1+C\ln\left(\dot{\varepsilon }/{\dot{\varepsilon }}_{0}\right)\right]$ and [1 − ((T − T_r_)/(T_m_ − T_r_))^m^]), degenerate to 1. Expression (1) is transformed into the following:

(2) $$\sigma =\left(A+B\varepsilon^{n}\right)$$

Here, *σ* represents the true stress, *ε* represents the true plastic strain, and *A* and *B* are material parameters that need to be determined through experiments. Rearranging Equation (2), we obtain the following:

(3) $$\ln\left(\sigma -A\right)=n\ln\varepsilon +\ln B$$

Here, *A* represents the yield strength of the material, which corresponds to a residual plastic strain of 0.2%.

Equation (3) can be considered a linear function, in which *lnε* is the independent variable, $\ln\left(\sigma -A\right)$ is the dependent variable, *n* is the slope, and ln⁡B is the intercept. Through linear fitting, specific numerical values for *B* and *n* can be obtained.

**Figure 2:**
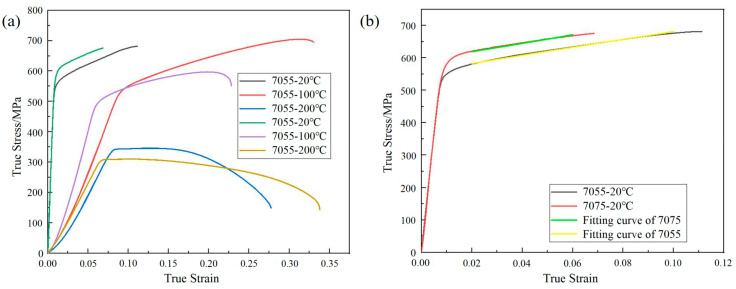
(**a**) True stress–strain curves for the 7055 and 7075 alloys under different temperature conditions; (**b**) fitting curves of parameters *B* and *n* for the 7055 alloy and 7075 alloy.

The true tensile stress–strain curves of 7055 and 7075 alloys under different temperature conditions are shown in Figure 2a. The values of *A* for the 7055 and 7075 alloys were determined to be 555 MPa and 595 MPa, respectively. The fitting curves for parameters *B* and *n* of the 7055 alloy and 7075 alloy are shown in Figure 2b. The obtained values of *B* and *n* for the 7055 alloy were 1097 and 0.94, respectively, while for the 7075 alloy, the values of *B* and *n* were 1421 and 1.04, respectively. The calculated value of parameter *m* for the 7055 alloy was 0.32, and the calculated value of parameter *m* for the 7075 alloy was 1.47.

SHPB experiments were conducted at room temperature and at different strain rates. Expression (1) was transformed into the following:

(4) $$\sigma /\left(A+B\varepsilon^{n}\right)=C\ln\left(\dot{\varepsilon }/{\dot{\varepsilon }}_{0}\right)+1,$$

(5) $$and\;here\;\varepsilon *=\dot{\varepsilon }/{\dot{\varepsilon }}_{0}$$

Equation (4) can be considered a linear function, in which $\ln\varepsilon^{*}$ is the independent variable, $\frac{\sigma }{A+B\varepsilon n}$ is the dependent variable, *C* is the slope, and 1 is the intercept. Through linear fitting, the numerical value for parameter *C* could be determined.

High-temperature tensile experiments were conducted at the reference strain rate and at different temperatures. Expression (1) was transformed into the following:

(6) $$\ln\left[1-\frac{\sigma }{A+B\varepsilon^{n}}\right]=mlnT*$$

(7) $$T*=\left(T-Tr\right)/\left(Tm-Tr\right)$$

Equation (6) can be considered a linear function, in which lnT* is the independent variable, $\ln\left[1-\frac{\sigma }{A+B\varepsilon n}\right]$ is the dependent variable, and *m* is the slope. Through linear fitting, the numerical value for parameter *m* could be determined.

**Figure 3:**
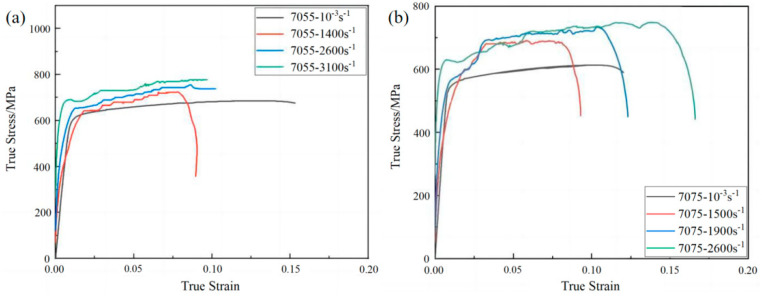
SHPB experimental results for (**a**) the 7055 alloy; (**b**) the 7055 alloy.

The results of the SHPB experiment for the 7055 alloy are shown in Figure 3a, and the results for the 7075 alloy are shown in Figure 3b. Based on the above analysis, the parameters of the J-C constitutive model for the 7055 and 7075 alloys are listed in Table 3.

### 3.2. J-C Damage and Fracture Model

To assess the fracture behavior of the materials, this study established damage equations for the J-C models of the two aluminum alloys. The parameter values used in the J-C model were determined through experiments. The expression for the J-C model was as follows [37]:

(8) $$\varepsilon_{f}=\left[D_{1}+D_{2}\exp\left(D_{3}\sigma *\right)\right]\left(1+D_{4}\;ln\varepsilon *\right)\left(1+D_{5}T*\right)\;$$

The J-C model incorporated parameters D1~D3, which are associated with stress triaxiality [40]. This study utilized tensile experiment bars with different notch radii for the 7055 and the 7075 alloys at reference strain rates. The experimental results could be evaluated as a function of the fracture performance of the two alloys.

Concerning fracture strain, Gambirasio et al. [41] proposed that the plastic volume of a metallic material remains consistent before and after the tensile experiment. The local fracture strain of the specimen can be computed by assessing the post-test cross-sectional area, as shown in the following equation:

(9) $$\varepsilon_{f}=\ln\left(A_{0}∕A_{f}\right)$$

Here, *A*_0_ represents the initial minimum cross-sectional area of the specimen, and $A_{f}$ denotes the minimum cross-sectional area at the point of fracture in the notched region. By measuring the minimum cross-sectional area before and after the test, the fracture strain for several experiments was calculated. The fracture strains of the 7055 and 7075 alloys are shown in Table 4.

The expression for stress triaxiality of rod-shaped tensile specimens is as follows:

(10) $$\eta =\frac{1}{3}+\ln\left(1+\frac{a_{0}}{{2R}_{0}}\right)$$

Here, a_0_ is the radius of the minimum cross-section of the pattern, and *R*_0_ is the radius of the notch of the pattern. Based on the fracture strain values obtained from Table 4 and the stress triaxiality values obtained through Expression 10, the *D*_1_~*D*_3_ values of the 7055 alloy were determined using the curve fitting method. Parameters *D*_1_~*D*_3_ of the J-C model for the 7055 alloy were 0.195, 0.670, and −3.900, respectively.

**Figure 4:**
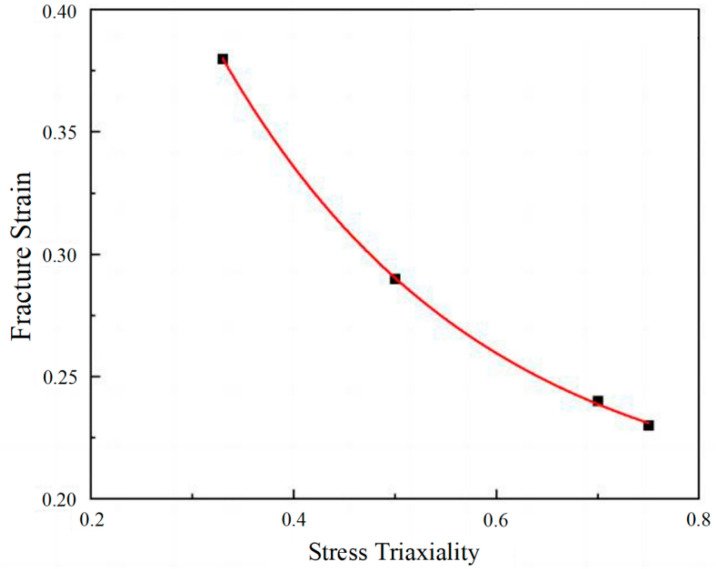
The fitting curve of the 7055 alloy.

Due to the limited correlation between fracture strain and stress triaxiality for the 7075 alloy in this experiment, fracture parameters of the 7075 alloy from the literature [42] were used. Figure 4 shows the fitting curves of J-C parameters *D*_1_ ~*D*_3_ for the 7055 alloy, with the fitting equation ε*_f_* = [0.195 + 0.670exp(−3.900σ∗)]. From the equation above, parameters *D*_1_~*D*_3_ could be obtained. The parameters of the J-C model obtained from the calculation are shown in Table 5.

### 3.3. Simulation of the Penetration Process

In order to assess the anti-penetration performance of the aluminum alloy laminate, numerical simulations were conducted using the ABAQUS explicit dynamics module.

**Figure 5:**
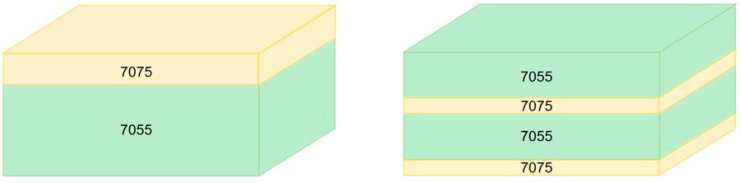
Laminated composite structure.

Models of the projectile and laminates were established. Eight laminate configurations were selected: 7075/7055, 7055/7075, 7055/7075/7055/7075, 7075/7055/7075/7055, 7055/7075/7075/7055, 7075/7055/7055/7075, 7055/7055/7075/7075, and 7075/7075/7055/7055. A schematic of the laminated composite structure is illustrated in Figure 5. The dimensions of the laminate were 100 mm × 100 mm × 36 mm, and the thickness ratio between the 7075 and 7055 layers was 1:5. Steel was selected as the projectile material, and the J-C constitutive model parameters from Table 3 and Table 5 were input into the software and assigned to each layer of the laminate. The projectile was coupled as a rigid body, and the lateral boundaries of the laminate were fixed, with a friction coefficient of 0.25. The initial velocity of the projectile along the negative *y*-axis was set at 900 m/s. In the experiment, the 7075 and 7055 alloys were combined using a hot rolling process, and a closed connection was employed at the contact surfaces between each layer of the laminate. It should be noted that the mechanical properties of the hot-rolled bonding interface have not been systematically characterized in this study, which may have led to potential deviations in evaluating the penetration resistance of the laminated plates.

Meshing was performed on both the projectile and the laminate, with a mesh size of 0.8 mm × 0.8 mm × 0.8 mm for the projectile and 0.7 mm × 0.7 mm × 0.7 mm for the laminate. A C3D8R was used to divide the mesh when building the laminates. Additionally, a circular region centered on the point of projectile penetration, with a radius twice that of the projectile radius, was finely meshed with a mesh size of 0.25 mm × 0.25 mm × 0.25 mm. Since the projectile tended to penetrate the interior of the laminate, a node set containing all the nodes of the laminate was established.

**Figure 6:**
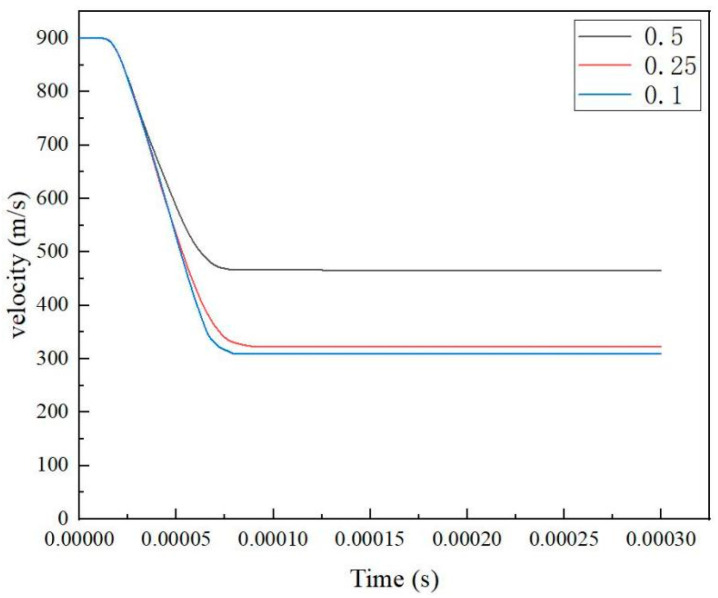
Variation in projectile velocity under different mesh sizes.

To ensure the accuracy of the calculation results was not affected by mesh size, we conducted an analysis of the laminated plate model with different mesh sizes. Specifically, simulations were performed using mesh sizes of 0.1 mm, 0.25 mm, and 0.5 mm. This approach eliminated the potential impacts of mesh size on the results, and allowed for accurate evaluation of computational efficiency and precision. Table 6 presents the residual velocities of the bullet under different mesh sizes, and Figure 6 illustrates the bullet velocity variations. The results demonstrate that when the mesh size was ≤0.25 mm, the calculation results converged to consistent values. However, compared to smaller mesh sizes, the 0.25 mm mesh achieved a shorter computation time, while ensuring reliable accuracy, significantly improving work efficiency.

**Figure 7:**
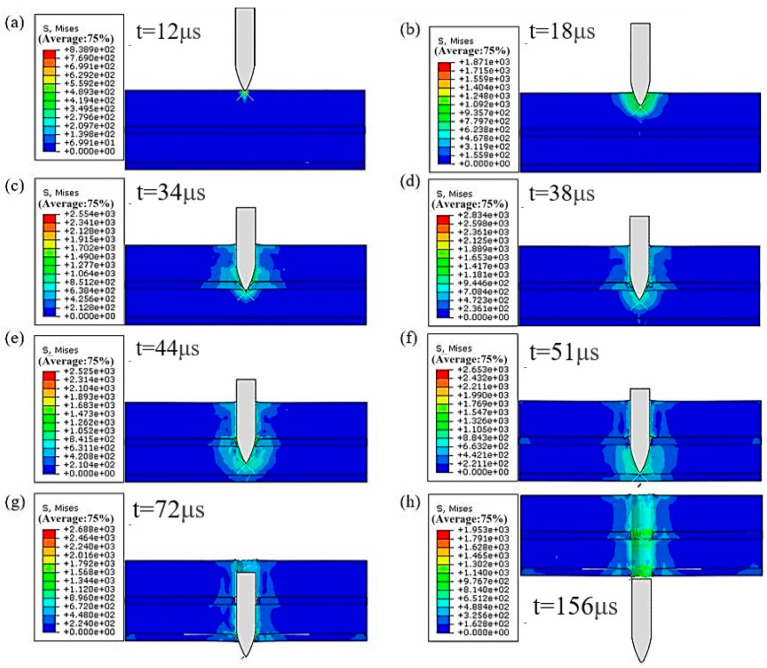
Bullet penetration process.(**a**) early stage of penetrating the first layer; (**b**) middle stage of penetrating the first layer; (**c**) early stage of penetrating the second layer; (**d**) later stage of penetrating the second layer; (**e**) middle stage of penetrating the third layer; (**f**) later stage of penetrating the third layer; (**g**) bullet completely penetrates the laminate; (**h**) bullet completely penetrates the laminate.

**Figure 8:**
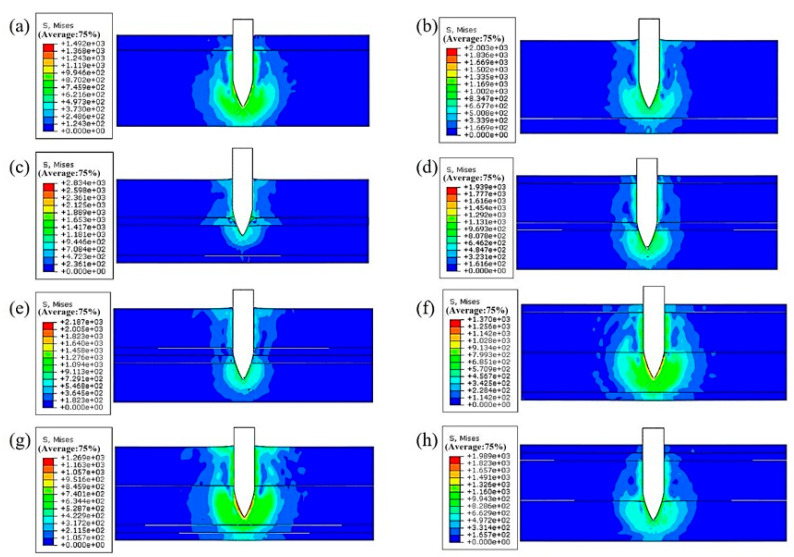
A penetration schematic of laminates with different configurations at the same time instant: (**a**) 7075/7055; (**b**) 7055/7075; (**c**) 7055/7075/7055/7075; (**d**) 7075/7055/7075/7055; (**e**) 7055/7075/7075/7055; (**f**) 7075/7055/7055/7075; (**g**) 7055/7055/7075/7075; (**h**) 7075/7075/7055/7055.

During the establishment of surface-to-surface contact, the master surface was chosen as the surface containing the projectile, and the slave surface was chosen as the node set. The results of the bullet penetration process with an initial velocity of 900 m/s are shown in Figure 7. The penetration for laminates with different configurations is shown in Figure 8.

**Figure 9:**
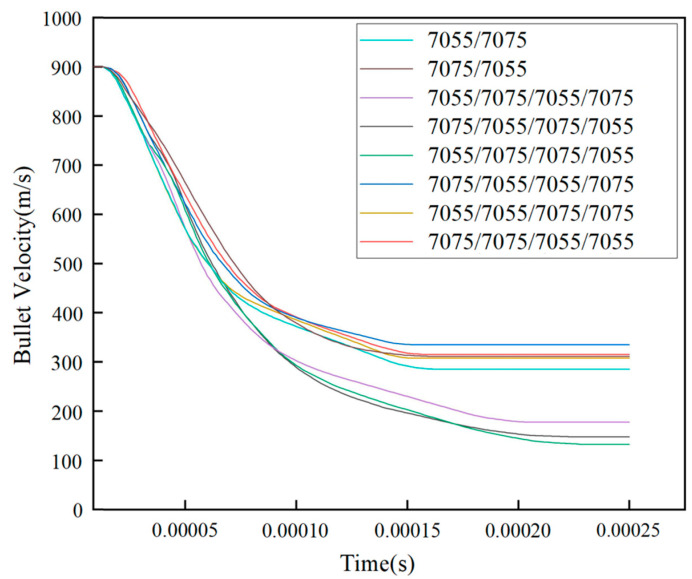
Bullet velocity curves for different laminate configurations.

Table 7 shows the residual velocities of bullets with different initial velocities for the 7055/7055/7075/7075 configuration. Figure 9 shows the change in velocity of the bullet for the eight types of laminate configuration. The bullet tip is adopted as the reference point for velocity measurement. The analysis indicates that, at the same initial bullet velocity, the 7055/7075/7075/7055 configuration exhibits the lowest remaining bullet velocity, indicating optimal anti-penetration performance for this configuration.

As shown in Figure 7, due to the bullet’s sharp geometry, a high-stress zone is created instantaneously upon contact with the laminated plate, causing minor protrusion at the laminate’s top surface and initiating damage on the front surface. As the bullet progresses through the plate, the high-stress zone around it expands progressively, accompanied by significant plastic deformation and material fracture near the bullet hole. This leads to the elimination of numerous failure elements, a conclusion further corroborated in the literature [38]. During the entire process of bullet penetration, the bullet’s kinetic energy gradually converts into thermal energy, plastic deformation of the laminate, and the energy required for fracture.

The result indicates that when a bullet comes into contact with the layer interface, deformation and fracture of the layer interface consume the bullet’s kinetic energy, leading to a rapid decrease in its velocity. In Figure 9, when the bullet penetrates the laminated plate at an initial velocity of 900 m/s, the bullet exhibits rapid velocity decay during the 0–0.0001 s interval. This is primarily due to the significant stress concentration generated at the impact surface of the laminated plate, where a substantial portion of the bullet’s kinetic energy is consumed to overcome the plate’s resistance. As the bullet gradually penetrates more deeply, the resistance from the plate diminishes, leading to a slower velocity decay rate. During the 0.0001–0.00015 s period, the velocity decline becomes progressively gentler as the bullet tip penetrates further into the plate, further reducing the plate’s resistance and the work required to overcome it. By 0.00015–0.00025 s, laminated plates with configurations 7055/7075, 7075/7055, 7075/7055/7055/7075, 7055/7055/7075/7075, and 7075/7075/7055/7055 are fully penetrated, while configurations 7055/7075/7055/7075, 7075/7055/7075/7055, and 7055/7075/7075/7055 are penetrated after 0.0002 s, demonstrating their enhanced penetration resistance.

The simulation prediction results indicate that the anti-penetration performance of four-layer laminates may not necessarily be better than that of double-layer laminates, indicating that methods to improve the anti-penetration performance of laminates should not only increase the number of laminate layers, but also consider designing a reasonable laminate configuration. For 7055/7075 laminates, the optimal configuration predicted by the simulation was 7055/7075/7075/7055 when the thickness ratio of 7075 to 7055 was 1:5.

**Figure 10:**
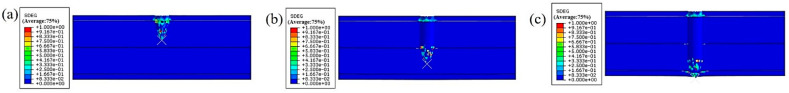
Contour plots of SDEG evolution. (**a**) SDEG distribution in early penetration phase, (**b**) SDEG distribution in mid-penetration phase, (**c**) SDEG distribution in terminal penetration phase.

In addition to variations in the bullet’s velocity, the damage and failure mechanisms of the laminated plates under bullet penetration should also account for material failure-related variables. Figure 10a–c illustrate the contour evolution of the stiffness degradation rate (SDEG) in elements around the perforation site during the bullet’s penetration. The SDEG values range from 0 to 1, where a higher value indicates more severe element damage. The critical failure threshold was set at 0.7, meaning that when the SDEG value approached 0.7, the corresponding mesh elements were considered to have failed, and were subsequently removed from the simulation.

## 4. Conclusions

(1) From the velocity curve for bullet penetration, it can be seen that the velocity of the bullet sharply decreases in the initial stage of penetration, but in the later stage, it slows down. This indicates that the strain and stress generated by the deformation of the front end of the laminate may cause local instability of the rear part of the material, which reduces the anti-penetration performance of the laminate.
(2) Numerical simulation was conducted on the bullet penetration process of 7055/7075 aluminum alloy laminates with different configurations, in order to evaluate their anti-penetration performance. The simulation results indicate that when the thickness ratio of 7075 and 7055 is 1:5, the optimal configuration of the composite plate is 7055/7075/7075/7055.

## Figures and Tables

**Table 1:** The chemical composition of the alloy (wt. %).

Alloy	Element Content								
	Si	Fe	Cu	Mn	Mg	Cr	Zn	Ti	Zr
7055	0.1	0.15	2.4	0.05	1.96	0.04	7.75	0.06	0.08
7075	0.4	0.5	1.8	0.3	2.4	0.1	5.8	0.1	—

**Table 2:** The meanings of all the parameters in the J-C model.

Parameters	Meaning
σ	Flow stress
ε	Current experimental strain rate
$\dot{\varepsilon }$	Reference strain rate
${\dot{\varepsilon }}_{0}$	Current temperature
T	Material melting point
$T_{m}$	Room temperature
$T_{r}$	Yield strength at reference
A	strain rate
B	Strain hardening factor
n	Strain hardening index
C	Factors related to strain rate reinforcement
m	Factors related to thermal softening

**Table 3:** The parameters of the J-C constitutive model for the 7055 and 7075 alloys.

Alloy	A	B	n	C	m
7055	555	1097	0.94	0.011	0.32
7075	595	1421	1.04	0.001	1.47

**Table 4:** The fracture strains of the 7055 and 7075 alloys.

Sample	Initial Minimum Cross-Sectional Diameter (mm)	Minimum Cross-Sectional Diameter After Fracture (mm)	Fracture Strain
7055-1	6.01	4.97	0.38
7055-2	4.06	3.64	0.23
7055-3	4.03	3.56	0.24
7055-4	4.16	3.58	0.29
7075-1	6.02	5.12	0.16
7075-2	4.06	3.49	0.17
7075-3	4.06	3.34	0.20
7075-4	4.09	3.37	0.19

**Table 5:** The fracture parameters of the J-C model.

Alloy	$D_{1}$	$D_{2}$	$D_{3}$	$D_{4}$	$D_{5}$
7055	0.195	0.670	−3.900	0.04	4.72
7075 [42]	−0.428	0.757	−3.408	−0.003	24.93

**Table 6:** Residual bullet velocity under different mesh sizes.

Mesh Size	Residual Velocity (m/s)	Computational Time (h)
0.5	466	0.2
0.25	322	0.45
0.1	309	1.5

**Table 7:** The remaining velocity of bullets with different initial velocities for the 7055/7 055/7075/7075 configuration.

Initial Velocity (m/s)	Residual Velocity (m/s)
800	0
850	81
900	225
1000	447

## Data Availability

The original contributions presented in this study are included in the article. Further inquiries can be directed to the corresponding author(s).
